# *Yersinia ruckeri* Infection and Enteric Redmouth Disease among Endangered Chinese Sturgeons, China, 2022

**DOI:** 10.3201/eid3006.231354

**Published:** 2024-06

**Authors:** Yibin Yang, Shijian Xu, Hao He, Xia Zhu, Yongtao Liu, Mou Hu, Bobin Jiang, Yuqiang Li, Xiaohui Ai, Guihong Fu, Hongyu Zhang

**Affiliations:** Key Laboratory of Sturgeon Genetics and Breeding, Ministry of Agriculture and Rural Affairs, Hangzhou Qiandao Lake Sturgeon Technology Co., Ltd., Hangzhou, China (Y. Yang, S. Xu, M. Hu, B. Jiang, Y. Li);; Yangtze River Fisheries Research Institute, Chinese Academy of Fishery Sciences, Wuhan, China (Y. Yang, H. He, X. Zhu, Y. Liu, X. Ai);; College of Animal Science and Technology, Hunan Agricultural University, Changsha, China (G. Fu);; Fishery Resource and Environment Research Center, Chinese Academy of Fishery Sciences, Beijing, China (H. Zhang)

**Keywords:** antimicrobial resistance, Chinese sturgeon, *Acipenser sinensis*, *Yersinia ruckeri*, enteric redmouth disease, endangered, species protection, disease prevention and control, bacteria, China

## Abstract

During October 2022, enteric redmouth disease (ERM) affected Chinese sturgeons at a farm in Hubei, China, causing mass mortality. Affected fish exhibited characteristic red mouth and intestinal inflammation. Investigation led to isolation of a prominent bacterial strain, zhx1, from the internal organs and intestines of affected fish. Artificial infection experiments confirmed the role of zhx1 as the pathogen responsible for the deaths. The primary pathologic manifestations consisted of degeneration, necrosis, and inflammatory reactions, resulting in multiple organ dysfunction and death. Whole-genome sequencing of the bacteria identified zhx1 as *Yersinia ruckeri*, which possesses 135 drug-resistance genes and 443 virulence factor-related genes. Drug-susceptibility testing of zhx1 demonstrated high sensitivity to chloramphenicol and florfenicol but varying degrees of resistance to 18 other antimicrobial drugs. Identifying the pathogenic bacteria associated with ERM in Chinese sturgeons establishes a theoretical foundation for the effective prevention and control of this disease.

Chinese sturgeons (*Acipenser sinensis*) are large migratory fish that are native to the Yangtze River and coastal areas of China (*1*). However, human activities (e.g., water-related engineering projects, fishing, pollution, and shipping) have degraded or destroyed much of the Chinese sturgeon natural habitat (*2*–*4*), limiting the suitable area for their reproduction (*5*) and resulting in a sharp decrease in natural population. Consequently, the Chinese sturgeon has been designated as a first-class protected animal in China (*2*). Since 2012, the Yangtze River Fisheries Research Institute has been conducting artificial breeding of Chinese sturgeons (*1*), establishing an artificial population, which is relevant to implementing large-scale reproduction and release activities of Chinese sturgeons and continuation of the species. However, artificial breeding poses some challenges to species preservation because of degradation of genetic resources, intensive cultivation practices, and the imbalance in nutritional requirements. Disease resistance among artificially reared Chinese sturgeons is low, and the fish are highly susceptible to pathogenic microorganisms such as *Aeromonas* (*6*), *Mycobacterium* (*7*), and *Pseudomonas* (*8*). Infections frequently result in large-scale mortality of Chinese sturgeons, imposing obstacles to their conservation.

In October 2022, a farm in Hubei, China, experienced a mass mortality event among artificially bred Chinese sturgeon offspring. To investigate the cause, we used pathogen isolation, pathology assessment, artificial infection, drug sensitivity testing, and bacterial whole-genome analysis.

All animal experiments were approved and conducted in compliance with the experimental practices and standards developed by the Animal Welfare and Research Ethics Committee of Yangtze River Fisheries Research Institute (YFI2022YYB019). The animals used in this study were derived from commercial sources, and owners’ consent was not required. All surviving fish continue to be cultured in the laboratory in accordance with standard breeding procedures.

## Materials and Methods

### Fish

We collected Chinese sturgeons displaying clinical signs and transported them to our laboratory (Yangtze River Fisheries Research Institute, Chinese Academy of Fishery Sciences, Wuhan, China) for disease diagnosis and isolation of pathogens. For infection experiments, we obtained healthy hybrid sturgeons (*Acipenser baeri* [male] × *Acipenser schrenckii* [female]) from farms without any history of such diseases. The hybrid sturgeons selected were energetic, showed no visible scars, and weighed 100 ±10 g. The hybrid sturgeons were temporarily housed in buckets for 7 days to confirm their health before infection testing.

### Pathogen Confirmation

We investigated the breeding environment and water source of the Chinese sturgeon farm to determine the temperature and water quality conditions during the onset of disease as well as the disease history and drug use at the farm. To identify the key characteristics of the disease, we examined the body surface and anatomy of Chinese sturgeons displaying typical signs and those in critical condition. Fish showing typical signs were brought back to the laboratory, where we examined the internal organs and gills under an optical microscope to look for parasites and fungi (*9*).

Initially, we anesthetized the Chinese sturgeons and placed them on ice and disinfected their entire body with 75% ethanol. Using an inoculation ring, we sampled blood, kidneys, and intestines of each fish and inoculated the samples onto brain–heart infusion agar plates, incubated at 28°C for 24 hours. We selected the dominant strain on the plate for further purification, resulting in a dominant strain temporarily named zhx1. To preserve the purified strain, we added 15% glycerol, mixed well, and then stored it at –80°C for future use (*10*,*11*).

We fixed intestines, spleen, liver, kidneys, and gills of the Chinese sturgeons that had displayed clinical signs with 10% neutral formalin fixative. We then prepared slides of the tissues and performed histologic examination according to standard methods (*12*,*13*). We inoculated the isolated strain zhx1 onto brain–heart infusion agar plates and cultured them at 28°C for 18 hours. We then washed the bacterial moss with sterile phosphate-buffered saline (PBS) and adjusted the bacterial suspension to different concentrations.

For the experiment with healthy fish, we randomly divided 150 healthy hybrid sturgeons into 5 group of 30 each: A, B, C, D, and E. Each sturgeon in groups A–D was injected with 0.1 mL of bacterial suspension at the base of the ventral fin, at concentrations of 10^9^ CFU/mL for group A, 10^8^ CFU/mL for group B, 10^7^ CFU/mL for group C, and 10^6^ CFU/mL for group D. The sturgeons in the control group (group E) were injected with an equal dose of sterile PBS at the same site. During the experiment, the fish were not fed, dissolved oxygen was maintained between 7.5 and 8.5 mg/L, the water temperature was controlled at 21°C to 22°C, and the fully aerated tap water was changed daily. We collected visceral tissues of dying sturgeons for bacterial isolation and purification and monitored the condition of the experimental hybrid sturgeons until deaths ceased. We recorded our observations of the experimental sturgeons daily and calculated the mortality rate.

### Antimicrobial Susceptibility of the Pathogen

We analyzed drug sensitivity of the zhx1 isolate by using the disk-diffusion method according to the Clinical and Laboratory Standards Institute antimicrobial drug sensitivity experimental standard (*14*). We inoculated the zhx1 isolate into brain–heart infusion and maintained the culture at 28°C with constant temperature oscillation (200 r/min) for 24 hours. Subsequently, we diluted the bacterial suspension with PBS to a concentration of 10^7^ CFU/mL. Next, we spread 100 μL of the bacterial suspension onto Mueller-Hinton agar plates, placed the drug-sensitive disks on the plates, incubated them at 28°C for 24 hours, and measured the diameter of the inhibition rings.

### Whole-Genome Sequencing

We extracted genomic DNA from bacterial cultures of zhx1 in brain–heart infusion by using a genomic DNA extraction kit (TaKaRa, https://www.takarabiomed.com). We used the PacBio Sequel platform (https://www.pacb.com) to sequence the entire genome. We used the hierarchical genome-assembly process version 2.3.0, single-molecular real-time analysis, for read assembly (*15*). We conducted predictions of coding DNA sequences by using Glimmer 3.02 (*16*). We generated the circular map of the genome by using Circos version 0.64 (*17*) and predicted genome islands by using the IslandPath-DIOMB genomic island prediction method (*18*). We predicted transfer RNA by using tRNAscan-Sev1.3.1 and ribosomal RNA by using Barrnap 0.7 software (*19*). We identified clusters of regularly interspaced short palindromic repeats by using MinCED (*20*). We performed functional annotation through a BLASTP search (BLAST 2.2.28+, https://blast.ncbi.nlm.nih.gov/Blast.cgi) against the National Center for Biotechnology Information nonredundant database, gene database, string database, and gene ontology (GO) database. Protein function classifications were based on clustering of protein homology group (clusters of orthologous genes) annotations by using the string database and BLASTP comparisons (*21*). We compared predicted genes with the Kyoto Encyclopedia of Genes and Genomes database by using the BLAST algorithm to identify corresponding genes involved in specific biologic pathways, based on the Kyoto Encyclopedia of Genes and Genomes orthogonal number obtained from the comparison (*22*). GO annotations were performed by using Blast2GO.

## Results

**Figure 1:**
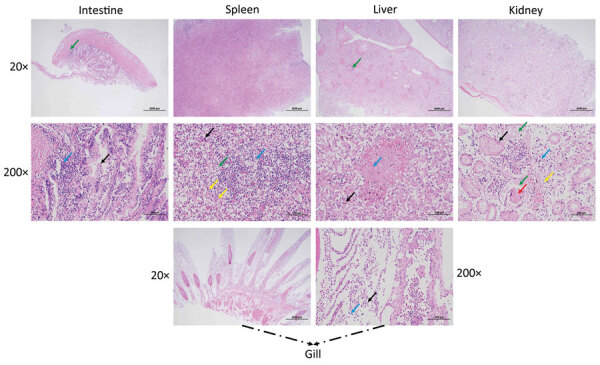
Pathologic changes in artificially bred Chinese sturgeon offspring infected with *Yersinia ruckeri*, China, 2022. A) Intestinal tissue showing mass intestinal villus necrosis and disordered structure; a large amount of mucosal epithelial cell necrosis and shedding (black arrow); extensive intestinal gland necrosis and connective tissue hyperplasia, with multiple lymphocyte infiltration (blue arrow); thick muscularis propria layer with small amount of connective tissue hyperplasia and small amount of lymphocyte infiltration (green arrow). B) Spleen tissue showing no obvious white pulp and unclear boundary between red pulp and white pulp; large area of red pulp congestion (black arrow); excessive lymphocyte necrosis and nuclear fragmentation (blue arrow); large number of lymphocytes with ballooning degeneration and vacuolization of the cytoplasm (green arrow); excessive infiltration of neutrophils (yellow arrows). C) Liver tissue showing diffuse balloon-like cell degeneration with vacuolization of cytoplasm (black arrow), dilation of liver sinuses, and irregular cell arrangement; multiple necrotic foci with a small amount of liver cell necrosis, nuclear lysis, and enhanced eosinophilia (blue arrow); small amount of focal infiltration of lymphocytes around the central vein (green arrow). D) Renal tissue showing diffuse watery degeneration of renal tubular epithelial cells with loose cytoplasm and light staining (black arrow) and loose arrangement of renal tubules; renal tubular epithelial cell necrosis, nuclear fragmentation (blue arrow), renal tubular structure disorder, accompanied by a small amount of lymphocyte infiltration (yellow arrow); excessive glomerular dilation with eosinophilic substances visible in the renal capsule (green arrow), cell necrosis and dissolution in the lumen, and disappearance of capillary loop structure (red arrow). E) Gill tissue showing small necrotic and detached pieces (black arrow) accompanied by a small amount of lymphocyte infiltration (blue arrow).

We identified pathologic changes in the intestines, spleen, liver, kidneys, and gills of infected Chinese sturgeons (Figure 1). Laboratory examination under microscopy did not reveal any parasitic or fungal infections. However, after bacteriological study, we isolated and identified a dominant bacterial strain, zhx.

**Figure 2:**
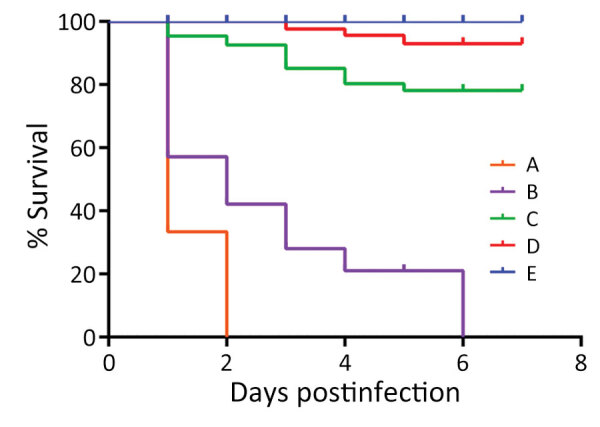
Survival rates for Chinese sturgeon experimentally inoculated with *Yersinia ruckeri* strain zhx1 isolated from artificially bred Chinese sturgeon offspring, China, 2022. Fish were injected with 0.1 mL of bacterial suspension at the base of the ventral fin at concentrations of 10^8^ (group A), 10^7^ (group B), 10^6^ (group C), or 10^5^ CFU/mL (group D) or with 0.1 mL phosphate-buffered saline as control (group E).

During the artificial infection and pathogenicity study, hybrid sturgeons in the experimental groups exhibited varying degrees of mortality (Figure 2). The mortality rate in groups A and B reached 100%, and the fish displayed signs similar to those that occur with natural onset (e.g., red mouth and enteritis), but red mouth appeared very late. In contrast, we observed no disease or death among fish in control group E. The same bacteria with morphologic characteristics similar to zhx1 were isolated from the dying hybrid sturgeons, and subsequent identification confirmed the bacteria to be the same: zhx1. The infection experiment followed Koch's postulates, indicating that zhx1 was the pathogenic bacteria causing Chinese sturgeon disease.

### Pathogen Confirmation

**Figure 3:**
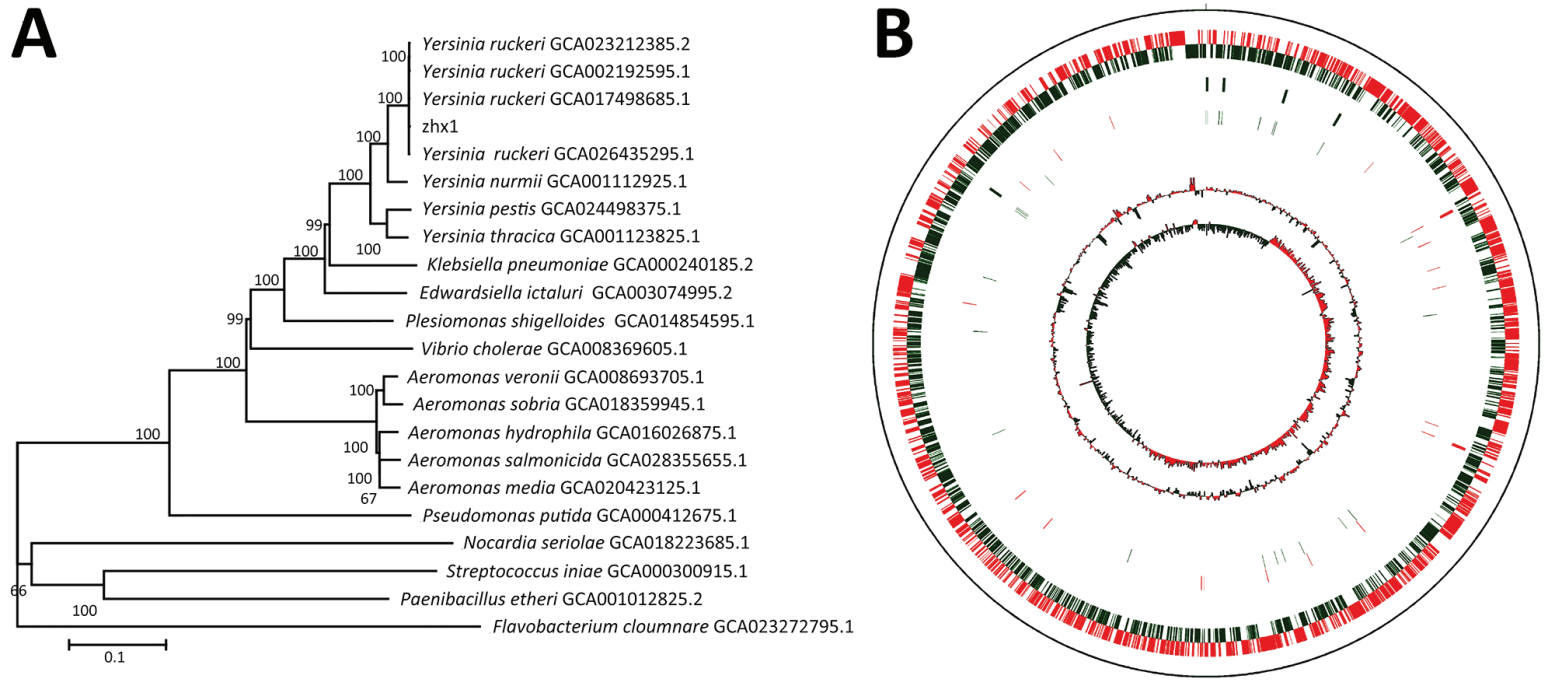
Genome characteristics of *Yersinia ruckeri* strain zhx1 isolated from artificially bred Chinese sturgeon offspring, China. A) Phylogenetic tree based on the whole genome of zhx1 and other pathogenic bacteria. B) Genome map of zhx1. The distribution of the circle from the outside indicates the genome size, forward coding DNA sequence (CDS), reverse CDS, repeat sequence, transfer RNA (black), ribosomal RNA (blue), and guanine-cytosine (GC) ratio. Colors indicate regions where the GC ratio is higher than average (red) and lower than average (green), and GC skewed either positive (red) or negative (green).

A phylogenetic tree based on bacterial whole-genome sequencing results (Figure 3, panel A) revealed clustering of zhx1 with *Y. ruckeri*, confirming that the isolated strain zhx1 is *Y. ruckeri*. The whole genome of zhx1 consists of a circular chromosome spanning 3,772,850 bp with an average guanine-cytosine content of 47.61% (Figure 3, panel B). Genome DNA sequencing generated 855,183 reads totaling 8,513,300,630 bp; sequencing depth was 1990×, and coverage rate was 100%. We identified 3,475 coding sequence genes, 22 ribosomal RNA genes, and 80 transfer RNA genes. Using the genomic island prediction method, we found 8 putative genomic islands in zhx1. In zhx1, we also identified 2 clustered regularly interspaced short palindromic repeats containing multiple short and repetitive sequences, 21-47–bp long. The sequencing reads are available in the National Center for Biotechnology Information Sequence Read Archive database (accession no. PRJNA1007872). 

The zhx1 genome consisted of 443 virulence factor-related genes (e.g., which affect hemolysin, flagella, enterobactin, and outer membrane protein A), 135 drug-resistance-related genes (e.g., which affect macrolide, fluoroquinolones, aminoglycosides, cephalosporins, tetracyclines, and phenicol), 109 carbohydrate enzyme-associated genes, and 514 host–pathogen interaction–associated genes.

### Pathogen Antimicrobial Susceptibility

We determined the sensitivity of the zhx1 isolate to 20 antimicrobial agents. zhx1 was highly sensitive to chloramphenicol and florfenicol and exhibited varying degrees of resistance to other drugs, especially those commonly used in aquaculture (e.g., doxycycline, neomycin) (Table).

**Table:** Antimicrobial susceptibility of *Yersinia ruckeri* strain zhx1 causing enteric redmouth disease in Chinese sturgeon, China, 2022*

Drug	Approximate judgment standard of inhibition zone, diameter, mm				Dose, μg	Inhibition zone diameter, mm, mean ± SD (susceptibility)
	R	I		S		
β-lactams						
Penicillin	<17		18–20	>21	10	0 (R)
Amoxicillin	<13		14–17	>18	20	0 (R)
Cephalosporins						
Ceftizoxime	<14		15–19	>20	30	0 (R)
Cefradine	<14		15–17	>18	30	0 (R)
Cefotaxime	<14		15–22	>23	30	0 (R)
Aminoglycosides						
Gentamicin	<12		13–14	>15	10	10.50 ± 0.50 (R)
Streptomycin	<11		12–14	>15	10	0 (R)
Netilmicin	<12		13–14	>15	30	9.00 ± 0.71(R)
Kanamycin	<13		14–17	>18	30	11.00 ± 1.41 (R)
Tobramycin	<12		13–14	>15	10	12.75 ± 0.83 (I)
Neomycin†	<12		13–16	>17	30	13.50 ±1.12 (I)
Macrolides						
Azithromycin	<13		14–17	>18	15	9.75 ± 1.09 (R)
Erythromycin	<13		14–22	>23	15	12.25 ± 0.83 (R)
Tetracyclines						
Tetracycline	<18		19–22	>23	30	18.00 ± 1.58 (I)
Doxycycline†	<12		13–15	>16	30	14.25 ± 1.48 (I)
Quinolones						
Enoxacin	<14		15–17	>18	10	14.75 ± 1.64 (I)
Norfloxacin	<12		13–16	>17	10	15.50 ± 1.12 (I)
Amphenicols						
Chloramphenicol	<12		13–17	>18	300	22.75 ± 0.83 (S)
Florfenicol†	<12		13–17	>18	75	20.75 ± 1.30 (S)
Sulfonamides						
Sulfisoxazole	<12		13–16	>17	300	11.25 ± 0.43 (R)

*I, intermediately sensitive; R, resistant; S, sensitive. †Veterinary antibiotics used in aquaculture.

## Discussion

**Figure 4:**
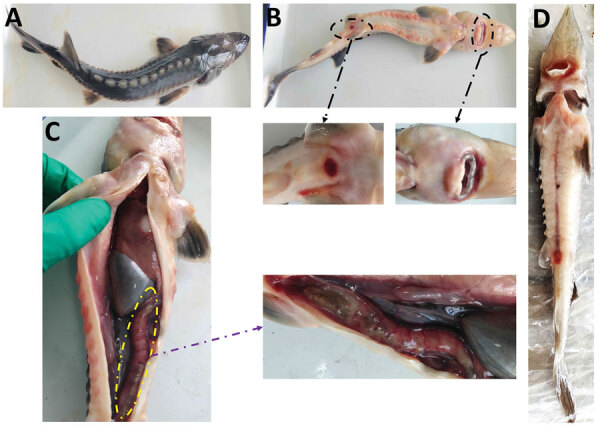
Clinical signs of Chinese sturgeons infected with *Yersinia ruckeri*, China. A–C) Naturally infected artificially bred Chinese sturgeon offspring: A) back; B) anal redness and swelling, red mouth; C) severe intestinal inflammation. D) Fish experimentally infected with *Y. ruckeri*.

Chinese sturgeon farms have been experiencing disease outbreaks of enteric redmouth disease (ERM) for many years; incidence rates are high. In some ponds, the incidence rate has reached 80% and the mortality rate 50%. The disease is observed in sturgeons weighing 1–2 kg, with no noticeable individual differences. Disease onset occurs at water temperature <22°C. Diseased Chinese sturgeons exhibit signs such as red mouth and severe enteritis (Figure 4).

*Y. ruckeri* is classified as family *Enterobacteriaceae*, genus *Yersinia*, and is the pathogen responsible for ERM in cold-water salmonid fish (*23*,*24*). ERM was first detected in rainbow trout in the United States in 1952; subsequently, the pathogen was isolated from infected rainbow trout by Ross et al. in 1965 (*23*). During the 1970s and 1980s, *Y. ruckeri* spread from the United States to Europe, primarily between the United Kingdom and the European continent, infecting wild and farmed salmon in freshwater and seawater (*25*,*26*). However, since ERM was initially reported, its host range and geographic distribution have gradually expanded. Of note, channel catfish (*Ictalurus punctatus*) have become a major target of *Y. ruckeri* infection, especially in China (*27*,*28*), resulting in considerable economic losses for the global aquaculture industry.

Although local fish farmers have noted ERM in Chinese sturgeons in the past, infections with *Y. ruckeri* were not investigated or reported, but Shaowu et al. reported it in Amur sturgeons (*Acipenser schrencki*) (*29*). In various species of infected fish, the main clinical signs of ERM are subcutaneous hemorrhage around the mouth, varying degrees of hemorrhage in multiple visceral organs, intestinal inflammation accompanied by yellow mucus, and similar signs (*24*,*28*). In our study, the diseased Chinese sturgeons showed clinical signs and pathologic changes consistent with signs characteristic of ERM (*24*,*28*). Disease coincided with water temperatures <22°C, consistent with the environmental requirements of *Y. ruckeri* (*30*). Furthermore, our histopathologic results confirmed that the disease in Chinese sturgeons followed the general pattern of *Y. ruckeri* infection (i.e., the pathogen invades the body through the circulatory system after entering either the gills or intestine) (*28*). Our results indicate expanded host range of *Y. ruckeri* infection. Other research has isolated *Y. ruckeri* pathogens from human infected wounds, suggesting its potential as a pathogenic bacterium for humans and other animals (*31*) and raising concerns regarding public health.

As first-class protected animals in China, Chinese sturgeons have been endangered, and their protection is of great value (*1*). The large-scale mortality caused by the infection of *Y. ruckeri* in Chinese sturgeons poses a threat to survival of the species, which may be catastrophic for the fish, creating significant challenges for species protection. Current prevention and control of bacterial diseases in fish rely mainly on antibacterial drugs (*32*). However, antibacterial drug use has resulted in bacterial drug resistance, which has induced numerous problems and garnered more attention (*33*). Our results indicate that zhx1 is a strain of severely drug-resistant *Y. ruckeri* bacteria. Genomewide analysis also revealed the presence of 133 drug-resistant genes in the chromosomal region of zhx1 (e.g., affecting macrolides, fluoroquinolones, aminoglycosides, cephalosporins, tetracyclines, and phenicol). However, carrying drug-resistance genes did not necessarily result in corresponding drug-resistant phenotypes (*34*). For example, zhx1 carried phenicol-resistant genes but demonstrated high sensitivity to chloramphenicol. Therefore, the mechanism of bacterial drug resistance is complex (*35*).

In conclusion, our findings contribute valuable insight for promoting healthy breeding, preventing disease, and protecting Chinese sturgeons. Selecting appropriate antimicrobial agents for treating ERM in Chinese sturgeons is challenging. Thus, the preferred approach has become using vaccines to prevent ERM. The formalin-inactivated vaccine of *Y. ruckeri* has shown promising results for preventing and treating salmonid fish diseases (*36*). Consequently, vaccination has become a practical method for preventing *Y. ruckeri* disease in fish (*37*). However, in China, because of strict restrictions on the use of biological products and rigorous reviews, no *Y. ruckeri* vaccine is currently available, which brings about substantial difficulties for prevention and control of ERM in Chinese sturgeons and becomes a bottleneck problem for protecting the species. Therefore, in-depth research on the mechanism of ERM in Chinese sturgeons focuses on the development of an inactivated vaccine against *Y. ruckeri*, and breakthroughs in administrative approval are crucial. Those actions will help overcome challenges associated with the prevention and control of ERM in Chinese sturgeons, safeguard the Chinese sturgeon species, and contribute to biodiversity.
